# A Cu(I)-Based MOF with Nonlinear Optical Properties and a Favorable Optical Limit Threshold

**DOI:** 10.3390/nano15020145

**Published:** 2025-01-20

**Authors:** Jing Cui, Zhaohui Yang, Yu Zhang, Zhaoxuan Fan, Jianquan Wang, Xiaoyun Qin, Lijun Gao, Haoran Yang, Shuangliang Liu, Liming Zhou, Shaoming Fang, Zhen Zhang

**Affiliations:** 1 Key Laboratory of Surface & Interface Science of Henan Province, Department of Material and Chemical Engineering, Zhengzhou University of Light Industry, Zhengzhou 450002, China; jingcui@zzuli.edu.cn (J.C.); yangzhaohui077@163.com (Z.Y.); xyqin@zzuli.edu.cn (X.Q.); gljsuzanne@163.com (L.G.); yanghr@zzuli.edu.cn (H.Y.); liushuangliang@zzuli.edu.cn (S.L.); mingfang@zzuli.edu.cn (S.F.); 2 Key Laboratory of Organic Integrated Circuit, Tianjin Key Laboratory of Molecular Optoelectronic Sciences & Ministry of Education, Department of Chemistry, School of Science, Tianjin University, Tianjin 300072, China

**Keywords:** coordination polymer, nonlinear optics, optical limiting, Z-scan

## Abstract

The exploitation of high-performance third-order nonlinear optical (NLO) materials that have a favorable optical limit (OL) threshold is essential due to a rise in the application of ultra-intense lasers. In this study, a Cu-based MOF (denoted as Cu-bpy) was synthesized, and its third-order NLO and OL properties were investigated using the Z-scan technique with the nanosecond laser pulse excitation set at 532 nm. The Cu-bpy exhibits a typical rate of reverse saturable absorption (RSA) with a third-order nonlinear absorption coefficient of 100 cm GW^−1^ and a favorable OL threshold of 0.75 J cm^−2^ (at a concentration of 1.6 mg mL^−1^), which is lower than that of most NLO materials that have been reported on so far. In addition, a DFT calculation was performed and was in agreement with our experimental results. Furthermore, the mechanism of the third-order NLO properties was illustrated as one-photon absorption (1PA). These results investigate the relationship between the structure and the nonlinear optical properties of Cu-bpy, and provide an experimental and theoretical basis for its use in optical limiting applications.

## 1. Introduction

NLO materials have received considerable attention due to an increase in their applications in a range of contexts, including optical data storage, optical communications, optical switching, and image-processing [1,2]. Optical limit (OL) devices based on nonlinear optical materials can effectively attenuate the intensity of laser transmissions, bringing them to a safer level while still transmitting low ambient light. They therefore have great application potential in protecting human eyes and optical devices from laser damage [3]. A variety of materials, such as fullerene (C_60_), semiconductor nanoparticles, quantum dots, porphyrins, and metalphthalocyanine, have been found to exhibit outstanding nonlinear optical properties [4]. Among them, Metal-Organic Frameworks (MOFs)—a class of porous materials with periodic network structures in one, two, or three dimensions formed by metal ions or metal clusters and organic ligands connected by ligand bonds according to a certain ratio and spatial structure [5,6,7]—have great application prospects in fields such as catalysis, sensing, nonlinear optics, and drug delivery [8,9,10,11].

The coordination between metal ions and organic ligands and the reduction of non-radiative transitions for MOFs due to the conformational limitations of organic units all lead to an improvement in the photophysical behavior [12,13]. Therefore, MOFs are considered to be the most promising materials for the development of OLs and frequency conversion applications. The NLO properties of MOF materials are closely related to their coordination metal ions, organic ligands, and topological structures [14]. On the one hand, the introduction of metal ions enhances the excited state absorption of organic molecules, thereby reducing the light-limiting threshold and improving their OL ability [15]. On the other hand, the ligands and metal ions in MOFs interact with their surroundings through a large number of strong and weak forces (ligand bonding, π-stacking, and hydrogen bonding), and this complex molecular environment will affect the electron density and polarizability of the system, which directly affects the third-order nonlinear properties [16]. Among the various MOF materials, Cu-based MOFs are one of the most studied types. The stability of copper ions in aqueous solutions and their high affinity for organic ligands make the synthesis of Cu-based MOFs relatively easy. In addition, copper ions introduce unique optical and electronic properties into MOFs, such as photoluminescence and photoelectrochemical activity. Chen et al. [17] studied the influence of the metal ion on NLO properties. The intramolecular delocalization of π electrons and charge transfer due to the conjugated system formed by the *d-π* interaction greatly enhance the nonlinear optical properties [18,19,20,21], resulting in a nonlinear optical coefficient of 5.2 × 10^−10^ m W^−1^. Liang et al. [22] found that, with the picosecond laser excitation, the increase of coordination amounts can realize the transformation from saturable absorption (SA) to reverse saturable absorption (RSA).

As a rigid bridging ligand, the N atoms at both ends of the 4,4′-bipyridine (bpy) compound can simultaneously bridge different metal atoms due to their good coordination properties. The 4,4′-bipyridine compound gives its bridged complexes a variety of configurations due to the arbitrary rotation of the C-C single bond between the pyridine rings. In addition, the rigid planar conjugated structure can self-assemble into larger-scale supramolecular complexes through π-π stacking and hydrogen bonding [23]. However, there have been few reports on the nonlinear optical properties of polymers formed by the coordination of Cu-MOF with 4,4′-bipyridine.

In this study, we synthesized a Cu-based MOF (denoted as Cu-bpy) with 4,4′-bipyridine (bpy) as the ligand and copper ions as the coordination metal by a hydrothermal method and investigated the third-order NLO properties using the Z-scan technique. Cu-bpy in ethanol suspension (1.6 mg mL^−1^) showed the largest *β* values (100 cm GW^−1^) and the lowest OL threshold of 0.75 J cm^−2^, which is more competitive than has been demonstrated in previous reports. The mechanism behind the NLO properties of Cu-bpy was studied using first-principle calculations. Density Functional Theory (DFT) calculations were also performed to study the fundamental structure of Cu-bpy. Furthermore, Cu-bpy showed strong third-harmonic generation (THG).

## 2. Materials and Methods

The materials and chemical reagents used in this study are all provided in the S1 Section (see the Supplementary Materials section).

### 2.1. The Synthesis of Cu-Bpy

The Cu-based MOF was synthesized according a previous publication [23] but with modifications [24]: 186 mg Cu(ClO_4_)_2_·6H_2_O (0.5 mmol) and 192 mg 4,4′-bipyridine (1 mmol) were mixed in 18.0 mL of deionized water. After ultrasonication for 20 min, the system was transferred to a 25 mL Teflon reactor sealed with steel and heated to 180 °C for 72 h, then cooled to 30 °C at a rate of 2 °C h^−1^. After being washed three times with water, the resulting orange crystals were dried in a vacuum at 80 °C (denoted as Cu-bpy). (Caution: care should be taken after adding Cu(ClO_4_)_2_·6H_2_O, as an explosion may occur in the Teflon reactor during heating.)

### 2.2. The Z-Scan Technique

The NLO property of Cu-bpy was carried out using the Z-scan technology at room temperature. To do this, Cu-bpy was ground into powder and dispersed in ethanol at concentrations of 0.2 mg mL^−1^, 0.4 mg mL^−1^, 0.8 mg mL^−1^, and 1.6 mg mL^−1^. After standing for 30 min, the supernatant was collected for the Z-scan experiments. A Nd:YAG nanosecond laser (pulse 4 ns, repetition rate 10 Hz) with a wavelength of 532 nm was used as the excitation light source. As shown in Figure S3, the sample (Cu-bpy in a quartz cuvette) was placed onto a computer-controlled translation platform and moved symmetrically along the Z-axis (centered at zero). The nanosecond laser was split into two beams. One of the beams was reflected into detector 1 as the reference light, the other beam entered the sample through the focusing lens and was transmitted to detector 2.

## 3. Results

### 3.1. The Morphology and Structure of Cu-Bpy

**Figure 1:**
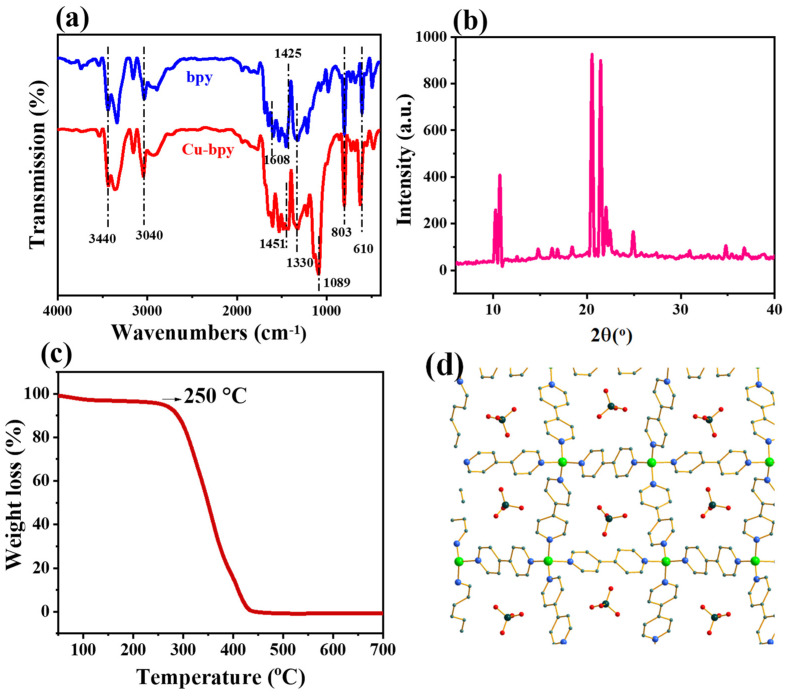
(**a**) Fourier transform infrared spectroscopy (FT-IR) spectra, (**b**) X-ray diffractograms, (**c**) TG curve of Cu-bpy, and (**d**) crystal structure diagram of Cu-bpy.

The FTIR spectra of Cu-bpy and 4,4′-bipyridine (for comparison) are shown in Figure 1a. For Cu-bpy, the absorption peak located at 3040 cm^−1^ is related to the stretching vibration of the C-H bond on the aromatic ring. The characteristic peak at 1330 cm^−1^ is assigned to the bending vibration of the C-N bond on the aromatic ring, while 1089 cm^−1^ is attributed to the C-O bond. The absorption peak at 803 cm^−1^ is related to the out-of-plane bending vibration of the aromatic hydrogen, and the peak at 610 cm^−1^ belongs to the in-plane deformation vibration of the pyridine ring. Cu-bpy shows diffraction peaks at 2θ = 10°, 2θ = 20°, and 2θ = 25° (Figure 1b) [24]. Thermal Gravimetric Analyzer (TGA) curves observed indicate that Cu-bpy has good thermal stability with an initial degradation temperature of 250 °C (Figure 1c). An analysis of single crystal structures (Figure S3 and Table S1) shows that Cu-bpy belongs to the monoclinic crystal system and the *C2/c* space group, consisting of a Cu^2+^ coordination with four pyridine rings. The perchlorate ion is also fixed in a repeating unit structure (Figure 1d), which is consistent with results found in previous reports [23].

**Figure 2:**
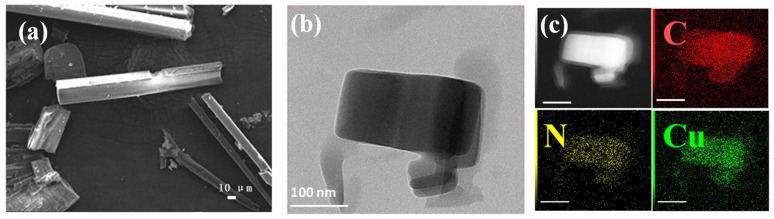
(**a**) SEM images, (**b**) TEM images, and (**c**) elemental mappings: C (red), N (yellow), and Cu (green) of Cu-bpy.

The morphology of Cu-bpy was investigated via scanning using an electron microscope (SEM) and a high-resolution transmission electron microscope (HR-TEM). The SEM images obtained show that Cu-bpy forms micrometer-sized rod-like crystals with widths ranging from 100 to 500 nm. The HR-TEM images (Figure 2b) showed that the grinded Cu-bpy took the form of a rectangular structure with a range of 100 to 200 nm after ultrasonication. EDX mapping (Figure 2c) showed that the elements C, N, and Cu were uniformly distributed in Cu-bpy.

**Figure 3:**
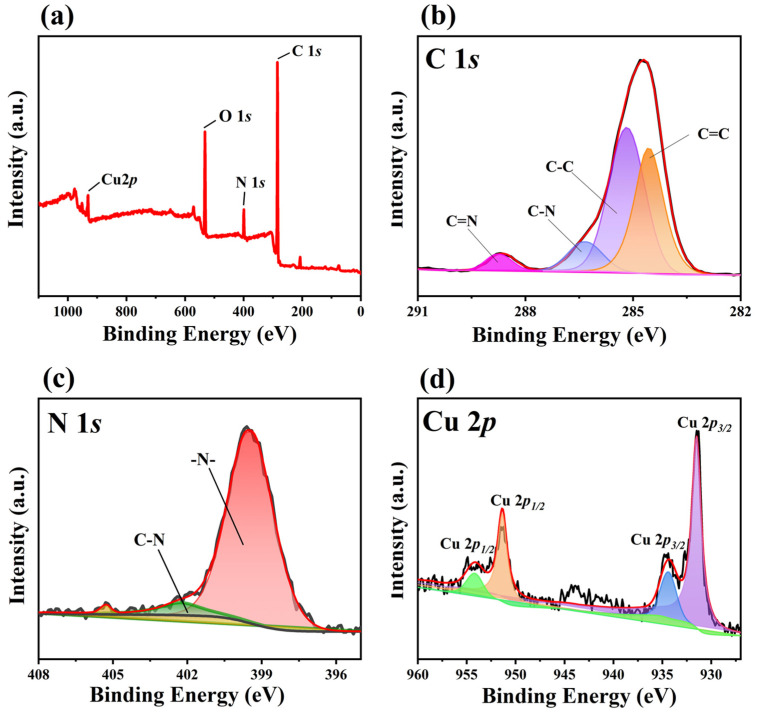
XPS spectra of Cu-bpy: (**a**) full spectrum, high-resolution XPS spectra of (**b**) C 1*s*, (**c**) N 1*s,* and (**d**) Cu 2*p*.

The chemical composition and surface chemical states of Cu-bpy were analyzed by XPS and the data were corrected with the binding energy of adsorbed carbon (284.8 eV). The XPS survey spectra (Figure 3a) showed that Cu-bpy exhibits characteristic peaks with binding energies (BEs) at 285 eV, 399 eV, 531 eV, and 931 eV, corresponding to C 1*s*, N 1*s*, O 1*s*, and Cu 2*p* signals, respectively [25]. The high-resolution spectrum of C 1*s* shows two experimental peaks around 284.8 eV and 286 eV, and has been divided into four characteristic peaks after fitting using Advantage software (Figure 3b), with BEs of 284.6 eV, 285.2 eV, 287.1 eV, and 288.8 eV belonging to C=C, C-C, C-N, and C=N, respectively [26]. For the N 1*s* spectra (Figure 3c), the peaks at BEs of 399.5 eV and 403.3 eV correspond to the C-N and metal-ligand nitrogen on the pyridine group, respectively [27]. The high-resolution of Cu 2*p* (Figure 3d) contains four characteristic peaks with BEs of 954.5 eV, 951.4 eV, and 935.8 eV, and 931.4 eV, corresponding to Cu 2*p*_1/2_ and Cu 2*p3/2*, respectively [28]. Since the rigid planar conjugated structure of 4,4′-bipyridine facilitates electron delocalization, CuI species can be generated via a single electron transfer from the ligand 4,4′-bipyridine on the MOF to the CuII center in the MOF cavity, or from the Cu0 center to the ligand 4,4′-bipyridine [29,30].

### 3.2. The Third Nonlinear Optical Properties of Cu-Bpy

**Figure 4:**
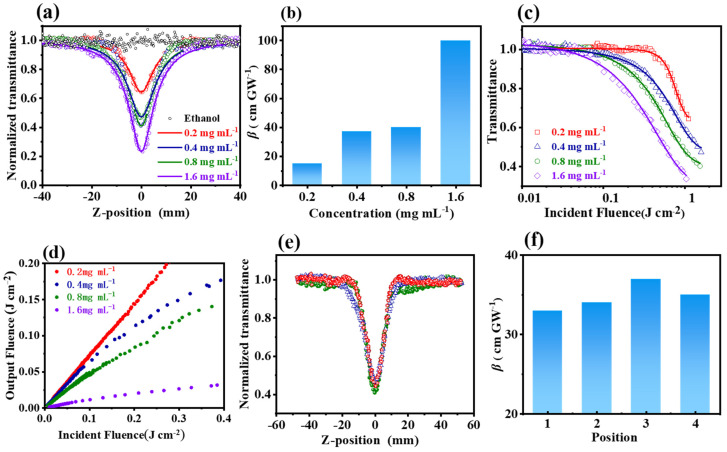
Nonlinear optical tests of Cu-bpy, Z-scan curve: (**a**) at different Cu-bpy concentrations with laser energy of 25 μJ; (**b**) variation of *β* values obtained by fitting Z-scan curves as a function of Cu-bpy concentration; (**c**,**d**) OL property of Cu-bpy with different concentrations; (**e**) Z-scan curves of Cu-bpy solution tested at random location of Cu-bpy solution (0.4 mg mL^−1^, 20 μJ); and (**f**) *β* values obtained from Z-scan curves of (**e**).

The nonlinear optical properties of Cu-bpy were investigated using the Z-scan technique with the 532 nm nanosecond laser [31]. Figure 4a shows the open-aperture (OA) Z-scan experimental results of Cu-bpy with different concentrations of ethanol. Before performing Cu-bpy, ethanol was first tested using Z-scan; the normalized transmittance did not change with the Z-axis position, indicating no nonlinear optical effects. However, Cu-bpy showed different curves from ethanol; the normalized transmittance varied with the movement of Cu-bpy. Take Cu-bpy with a concentration of 0.2 mg mL^−1^ (red line) as an example; when Cu-bpy moved from the initial position (*Z* = 40 mm) to the focal point (*Z* = 0), the transmittance gradually increased according to the incident light intensity. The normalized transmittance was kept constant (around 1.0), indicating the linear optical characteristic was at a low laser intensity. As Cu-bpy moved from Z = −10 mm towards the focal, the normalized transmittance gradually decreased with increasing laser intensity, indicating that the nonlinear effect had been activated. Moving further to Z = 40 mm, the normalized transmittance increased with Cu-bpy/focus distance. Therefore, the Z-scan curve of Cu-bpy showed a single-valley curve symmetry of focus, corresponding to the reverse saturation absorption (RSA) behavior.

Figure 4a shows the OA Z-scan curve of Cu-bpy at different concentrations under the constant input intensity (25 μJ). The normalized transmittance decreased with increasing Cu-bpy concentration from 0.65 (0.2 mg mL^−1^) to 0.22 (1.6 mg mL^−1^), indicating the uniform dispersion of the grinded Cu-bpy in ethanol.

To obtain the third-order nonlinear absorption coefficients (*β*), a theoretical fitting of the OA Z-scan data was performed [32]. The equation used was the following:

(1) $$T\left(z\right)=\frac{1}{\sqrt{\pi }q(z)}\int_{-\infty }^{+\infty }ln\left[1+q(z)exp(-\tau^{2})\right]d\tau$$

where $q_{0}=\frac{\beta I(0)L_{eff}}{{1+(z/z_{0})}^{2}}$, *I*(0) is the peak of the laser (532 nm) intensity at the focal, *T* is the normalized transmittance, *z* is the Rayleigh diffraction length of the laser beam calculated using $z=\pi \omega_{0}^{2}/\lambda$, where λ is the laser wavelength (532 nm) and *ω_0_* is the waist radius. In addition, $L_{eff}=\frac{1-e^{-\alpha L}}{\alpha }$ is the effective thickness of Cu-bpy, where *α* is the linear absorption coefficient and *L* is the thickness. The fitting results are shown by the solid lines in Figure 4a.

The third-order nonlinear absorption coefficient *β* values of Cu-bpy at different concentrations were extracted from Figure 4a and plotted as a function of Cu-bpy concentration (Figure 4b). As the Cu-bpy concentration increased, the *β* value increased accordingly. The *β* = 100 cm GW^−1^ was obtained for Cu-bpy. For example, the *β* value was two orders and two times larger than that of CH_3_NH_3_PbBr_3_ (Table 1).

Cu-bpy exhibits the RSA effect and hence can therefore be used for the development of OL devices. Figure 4c,d show the OL curves obtained by processing the OA Z-scan data. To do this, the energy density for each location was calculated according to Equations (2) and (3) below. The OL curve can then be obtained using the relationship of the incident energy density *F* as a function of the normalized nonlinear transmittance. That is:

(2) $$\omega (z)=\omega_{0}\sqrt{\left(1+(\frac{\lambda z}{\pi \omega_{0}^{2}}{)}^{2}\right)}$$

(3) $$F=\frac{E}{\pi \omega_{z}^{2}}$$

The OL curves of Cu-bpy at 0.2 mg mL^−1^, 0.4 mg mL^−1^, 0.8 mg mL^−1^, and 1.6 mg mL^−1^ are shown in Figure 4c and Figure 4d, respectively. For Cu-bpy with concentrations of 0.2 mg mL^−1^, 0.4 mg mL^−1^, and 0.8 mg mL^−1^ (see the red, blue, and green plots in Figure 4d), the output light intensity increased with the input laser intensity, but also deviated from linearity, indicating a weak OL effect at high intensity. The Cu-bpy with a concentration of 1.6 mg mL^−1^ showed a prominent OL effect. When the input laser intensity was weak, the output laser intensity increased linearly with the input intensity. When the input light intensity increased to about 0.7 J cm^−2^, the output laser intensity reached a threshold. With further increases to the input laser intensity, the OL effect was activated, restricting the output intensity to a certain level—i.e., at 0.04 J cm^−2^ in this work. This may be due to the fact that, at a low concentration (0.2 mg mL^−1^), Cu-bpy was independently dispersed in the ethanol. The RSA behavior was caused by the electron transfer between Cu(I) and 4,4′-bipyridine. When the concentration increased to 1.6 mg mL^−1^, Cu-bpy could increase, and a charge transfer occurred between Cu(I) and 4,4′-bipyridine in Cu-bpy or between Cu-bpy—i.e., the abundance of the π-penetrating framework interactions increased the electron delocalization/transfer, thus improving the OL performance [33]. The OL threshold (*Fth*), i.e., the energy density at which the transmittance is reduced to 50% of the linear transmittance, was calculated to be 0.75 J cm^−2^ for Cu-bpy (1.6 mg mL^−1^).

Compared to other published materials (Table 1), Cu-bpy shows a higher nonlinear absorption coefficient *β* and a lower OL threshold. This may be due to the following: (1) the arbitrary rotation of the C-C single bond between the pyridine rings, as 4,4′-bipyridine exhibits various configurations; (2) the π-π interaction can be formed by the pyridine ring in the Cu-bpy framework, or the adjacent pyridine between two independent Cu-bpy frameworks. Both of these can increase electron delocalization and transfer, thus improving the nonlinear optical response and achieving a high OL performance [33].

**Table 1:** Comparison of *β* values and OL threshold among different materials.

Materials	Timescale	Repetition Rate (Hz)	*β* (cm GW^−1^)	*F_th_* (J cm^−2^)	Ref.
CDGO	nanosecond	10	70	-	[34]
Mn(dnpi)_2_	nanosecond	10	2.3		[35]
ZnCu-MOF	nanosecond	10	44.7	-	[36]
CH_3_NH_3_PbBr_3_	nanosecond	400	8.6	-	[37]
MoS_2_-CuMOF	nanosecond	10	60		[38]
ZnTPyP(Cu)	nanosecond	5	5.7 × 10^5^	7.8	[39]
Zn_2_(TPyP)(AC)_2_	nanosecond	5	3.61 × 10^6^	0.32	[33]
Cu-bpy	nanosecond	10	100	0.75 ^a^	This work

[a] The optical limiting threshold for 1.6 mg mL^−1^ of Cu-bpy is 0.75 J cm^−2^.

The repeatability of the third-order nonlinear optical properties was tested using the Z-scan technique at random positions of the Cu-bpy ethanol suspension under the same experiment conditions (0.4 mg mL^−1^, 20 μJ). Similar RSA behavior with a normalized transmittance of 0.3~0.4 was observed (Figure 4f), and third-order nonlinear absorption coefficients of 33~37 cm/GW were obtained (Figure 4f).

**Figure 5:**
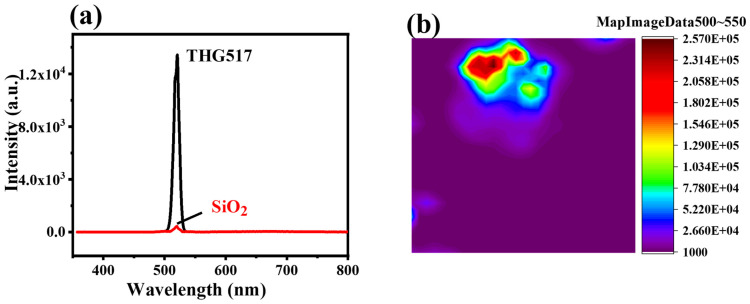
(**a**) THG spectrum and (**b**) THG intensity mapping of Cu-bpy excitation at 1550 nm.

The third harmonic generation (THG) of Cu-bpy was investigated using the harmonic measurement technique. A femtosecond laser with a wavelength of 1550 nm and a detection range of 300~900 nm was used. Figure 5a shows the THG signal intensity spectra of Cu-bpy; a prominent peak appears at 517 nm corresponding to 1/3 wavelength of the incident light of 1550 nm, manifesting the THG characteristics of Cu-bpy. The THG signal intensity of Cu-bpy is 24 times higher than that of SiO_2_. Figure 5b shows the THG intensity mapping of Cu-bpy. The harmonic intensity of Cu-bpy shows a strong signal throughout the crystal structure, which is expected to be applied in laser frequency conversion and in in vivo imaging.

### 3.3. The Mechanism of the NLO Properties of Cu-Bpy

**Figure 6:**
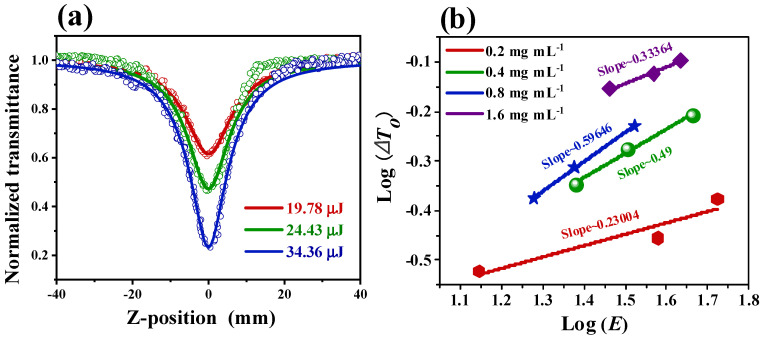
(**a**) *Z*-scan curves of Cu-bpy (0.8 mg mL^−1^) ethanol suspension solution with the laser excitation at 532 nm with different laser energies. (**b**) The relationship between Δ*T*_0_ and *E* in log-log scale corresponding to the 532 nm laser excitation.

To investigate the RSA behavior of Cu-bpy, the excited-state absorption cross section (*σ_ex_*) [40] of Cu-bpy was calculated as 6.45 × 10^−14^ cm^2^ by fitting the OA Z-scan curve. The ground-state absorption cross section (*σ_g_*) can be calculated by [41] using the following equation:

(4) $$\alpha =\alpha_{g}N_{A}C$$

where *C* is the molar concentration of Cu-bpy and *N_A_* is Avogadro’s number. The calculated *σ_g_* is 2.01 × 10^−15^ cm^2^. *σ_ex_* is larger than *σ_g_*, indicating that the main NLO procedure for Cu-bpy is RSA. Cu-bpy suspensions with concentrations of 0.2 mg mL^−1^, 0.4 mg mL^−1^, 0.8 mg mL^−1^, and 1.6 mg mL^−1^ were performed for OA *Z*-scan experiments with different pulse laser energy values, respectively (Figure 6a and Figure S4). The normalized transmittance curved with increases in laser energy. This is because more Cu-bpy MOFs were involved in the NLO response as the laser intensity increased [39]. The same tendency was found in other concentrations. The relationship between the changes of normalized transmittance (Δ*T*_0_) and the laser pulse energy (*E*) was plotted (Figure 6b). A good linear relationship can be observed for Δ*T*_0_ and *E* in the log-log scale. The linear fit of the Δ*T*_0_~*E* plot shows a slope of 0.2~0.5 for Cu-bpy with a concentration range of 0.2 mg mL^−1^ to 1.6 mg mL^−1^. The effective number of photons absorbed will be *n* + 1 [42,43], therefore the RSA behavior of Cu-bpy is probably one-photon absorption (1PA).

**Figure 7:**
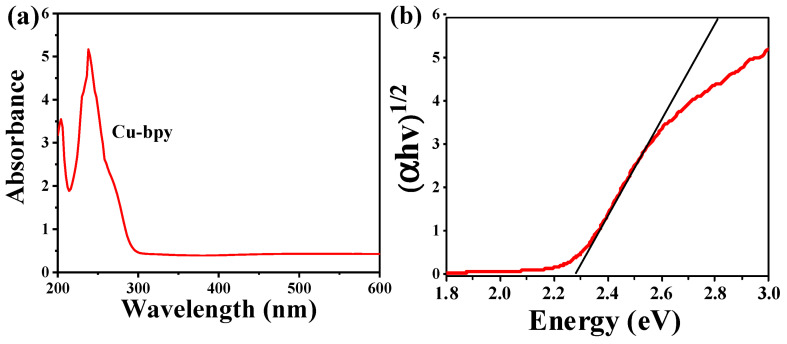
(**a**) Ultraviolet absorption diagram, and (**b**) band gap diagram of Cu-bpy.

To determine the absorption edge for Cu-bpy, a UV-visible absorption experiment was carried out. As shown in Figure 7a, Cu-bpy showed an adsorption peak of 260 nm, which could be due to the *d-d* electronic shifts of CuI cations, π-π* stacking of bpy ligands, or coordinated Cu centers acting on the excited state of bpy ligands. The band gap of Cu-bpy was estimated using the Davis-Mott formula, with the band relationship diagram of Cu-bpy shown in Figure 7b. The band gap of Cu-bpy was inferred to be 2.28 eV.

In addition, the fundamental electronic structure of Cu-bpy was calculated by first-principles density-functional theory (DFT) calculations (details of these are given in Section S6 of the ESM). Cu-bpy exhibits semiconducting behavior with a band gap of 2.39 eV (Figure S5), which is close to the experimental value of 2.28 eV.

**Figure 8:**
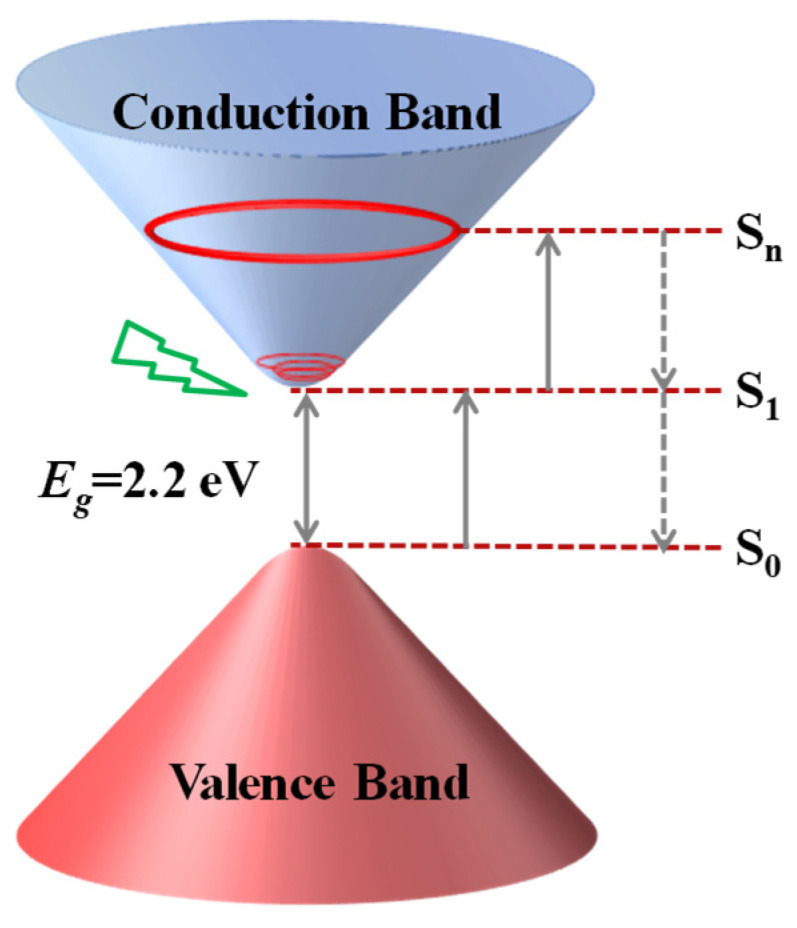
Schematic illustration of energy band for Cu-bpy.

To demonstrate the principle of the RSA and OL properties of Cu-bpy, the schematic energy band diagram for Cu-bpy is shown in Figure 8. The photon energy of the excited laser at 532 nm was calculated to be 2.33 eV, which is larger than the calculated energy gap (*E_g_*) of Cu-bpy (~2.28 eV); therefore, the absorption process was considered to be 1PA, which corresponds to the results derived from the slopes of Δ*T*_0_ and *E* in the log-log scale (Figure 6). When excited by the nanosecond laser at 532 nm, the ground state electrons of the Cu-bpy valence band (*S*_0_) absorbed one photon and jumped to the conduction band (*S*_1_) through an inter-band transition, resulting in the excited electron. The excited electron can then either return to the ground state (*S*_0_), or further in-band transition to *S_n_* by absorbing another photon, resulting in 1PA induced excited state absorption (ESA). Due to the short decay time between *S_n_* and *S*_0_ (picoseconds), the ESA on the nanosecond time scale does not deplete electrons at the *S*_1_ level [34], so more absorption can take place at the excited state, resulting in the RSA behavior of Cu-bpy.

## 4. Conclusions

In conclusion, a Cu-based MOF material Cu-bpy was synthesized. Cu-bpy belongs to monoclinic crystal structure with a C2/c space group. The third-order nonlinear optical properties of Cu-bpy were investigated using the Z-scan technique, and the RSA behavior was observed in Cu-bpy ethanol suspension solutions. The third-order nonlinear absorption coefficient was extracted as 100 cm GW^−1^, and the OL threshold was found to be 0.75 J cm^−2^ (1.6 mg mL^−1^), which is competitive compared with other Cu-based MOFs. Furthermore, Cu-bapy exhibits strong THG signal. These findings show that Cu-bpy is a potential NLO material for use in ultrafast photonic devices, optical limiting, and biosensing.

## Data Availability

Data are contained within the article and Supplementary Materials.

## Supplementary Materials

The following supporting information can be downloaded at: https://www.mdpi.com/article/10.3390/nano15020145/s1, Figure S1: The synthesis scheme of Cu-bpy; Table S1: Single crystal diffraction data of Cu-bpy material; Figure S2:Crystal structure diagram of Cu-bpy; Figure S3:The schematic of Z-scan measurement; Figure S4: Z-scan curve of Cu-bpy with concentraion of 0.2 mg mL^−1^, 0.4 mg mL^−1^, 1.6 mg mL^−1^ in ethanol. Figure S5:View of (a) HOMO and (b) LUMO for the profiles based on the DFT calculations of Cu–bpy.
