# Supplementary material for: A Cu(I)-Based MOF with Nonlinear Optical Properties and a Favorable Optical Limit Threshold
Source: Nanomaterials (Basel). 2025 Jan 20;15(2):145. doi: 10.3390/nano15020145 (PMC11767271; doi:10.3390/nano15020145)
Supplement: Supplementary file 1 [file nanomaterials-15-00145-s001.zip › nanomaterials-3431309-supplementary.pdf]

Supporting Information for

A Cu(I)-Based MOF with Nonlinear Optical Properties and a Favorable Optical Limit Threshold

Jing Cui [1], Zhaohui Yang [1], Yu Zhang [1], Zhaoxuan Fan [1], Jianquan Wang [1], Xiaoyun Qin [1], Lijun Gao [1], Haoran Yang [1], Shuangliang Liu [1], Liming Zhou [1,*], Shaoming Fang [1] and Zhen Zhang [2,*]

[1] Key Laboratory of Surface & Interface Science of Henan Province, Department of Material and Chemical Engineering, Zhengzhou University of Light Industry, Zhengzhou 450002, China; jingcui@zzuli.edu.cn (J.C.); yangzhaohui077@163.com (Z.Y.); xyqin@zzuli.edu.cn (X.Q.); gljsuzanne@163.com (L.G.); yanghr@zzuli.edu.cn (H.Y.); liushuangliang@zzuli.edu.cn (S.L.); mingfang@zzuli.edu.cn (S.F.)

[2] Key Laboratory of Organic Integrated Circuit, Tianjin Key Laboratory of Molecular Optoelectronic Sciences & Ministry of Education, Department of Chemistry, School of Science, Tianjin University, Tianjin 300072, China

* Correspondence: zlming1212@126.com (L.Z.); zhzhen@tju.edu.cn (Z.Z.)

Contents

Section S1 Materials and Methods

Section S2 Synthesis of Cu-BPY

Section S3 Crystal structure diagram of Cu-BPY

Section S4 Z-scan schematic diagram

Section S5 Z-scan test diagram of different concentration of Cu-BPY

Section S6 DFT calculations for the electronic structures of Cu–BPY

S1 Materials and Methods

S1.1 Materials

All the reagents and solvents employed were commercially available and were used as received without further purification. Copper perchlorate hexahydrate (analytically pure), 4,4'-bipyridine (analytically pure) were purchased from Shanghai McLean Biochemistry Technology Co.

S1.2 Basic Characterization

Powder X-ray diffraction (PXRD) Patterns was were collected using a D8Advance X-ray diffractometer using Cu−K_α radiation (λ = 0.1542 nm) with a scanning rate of 0.02° s^{-1}. FTIR data were recorded using a Bruker TENSORII FTIR(Bruker, Germany). X-ray photoelectron spectroscopy (XPS) spectra were obtained using an ESCALAB 250Xi instrument. Scanning electron microscope (SEM) images were taken using a JEOL JSM-7001F field-emission scanning electron microscope. Transmission electron microscope (TEM) images recorded were obtained via a JEM-2100 electron microscope. The UV–vis spectra were measured by Evolution201 spectrophotometer. Differential scanning calorimetry (DSC) curves were obtained using a TA Instrument Q2000 calorimeter with a heated/cooled a rate of 10 °C/min. Thermogravimetric analysis (TGA) was performed on a Perkin Elmer Pyris 1 (USA) under N_2 by heating to 850 °C at a rate of 10 °C min^{-1}.

S1.3 Density-functional theory calucation

The first-principles calculations were performed by the density-functional theory The first-principles calculations were performed by the density-functional theory (DFT), as implemented in plane-wave basis code Vienna ab initio simulation package (VASP). The ion-electron interactions were treated by the projector augmented wave (PAW)technique.Since the generalized gradient approximation in the Perdew– Burke–Ernzerhof (GGA-PBE) methods would underestimate the band gaps of semiconductor materials, the B3LYP functional with the dispersion correction were used to calculate the band gaps.

Section S2 Synthesis of Cu-bpy

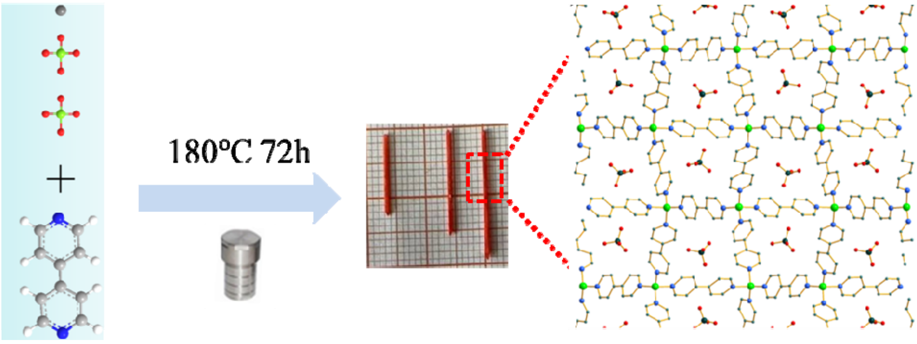

Figure S1 The synthesis scheme of Cu-bpy

Section S3 Crystal structure diagram of Cu-bpy

The crystal structure of Cu-bpy was tested and analyzed using a single crystal diffractometer of Agilent SuperNova, which was used to radiate graphite monochromized Mo *K*α and collect the data. The single crystal structure was analyzed by SHELXS program in Olex2 software, and the fine structure was modified by SHELXL program. Hydrogen atoms were added to the structure by geometric hydrogenation, refined by parameter self-adjusting model, and modified by full matrix least square method. All non-hydrogen atoms have been anisotropic refined. The determination of the chemical formula is completed by integrating crystallographic data, elemental analysis and thermogravimetric analysis. The structure and detailed data of Cu-bpy (Figure S3 and Table S1). Cu-bpy consists of a Cu^{2+} coordination with four pyridine rings, and the perchlorate ion is fixed in a repeating unit structure.

The analysis of single crystal structure shows that Cu-bpy belongs to monoclinic crystal system and *C2/c* space group. Cell parameters: *a*=7.1071 Å, *b*=33.0754 Å, *c*=17.5154 Å, α=90°, β=98.947°, γ=90°, *V*=4067.3 $Å^3$.

Table S1 Single crystal diffraction data of Cu-bpy material

	Cu-bpy
Empirical formula	$C_{80}H_{64}Cu_4N_{16}$,4(ClO_4)
M, g mol^{-1}	1901.47
Crystal system	Monoclinic
Space group	*P*2(1)/*c*
a, Å	7.1071(2)
b, Å	33.0754(9)

c, Å	17.5154(5)
α, deg	90
β, deg	98.947(3)
γ, deg	90
V, Å^3	4067.3
Z	2
D_{calcd}, g·cm^{-3}	1.553
T, K	286
μ, mm^{-1}	3.034
$R_1{}^a$ (I > 2σ(I))	0.0746
$wR_2{}^b$ (I > 2σ(I))	0.02024
$R_1{}^a$ (all data)	0.1063
$wR_2{}^b$ (all data)	0.2386
Diff peak and hole	1.392/0.527

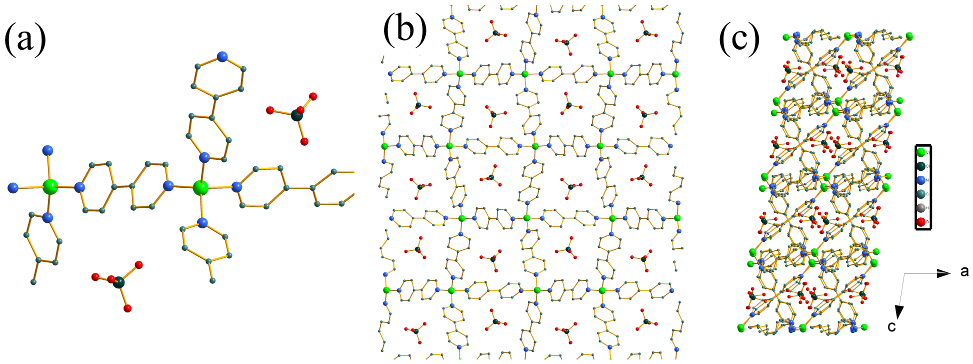

Figure S2 Crystal structure diagram of **Cu-bpy**

Section S4 Z-scan schematic diagram

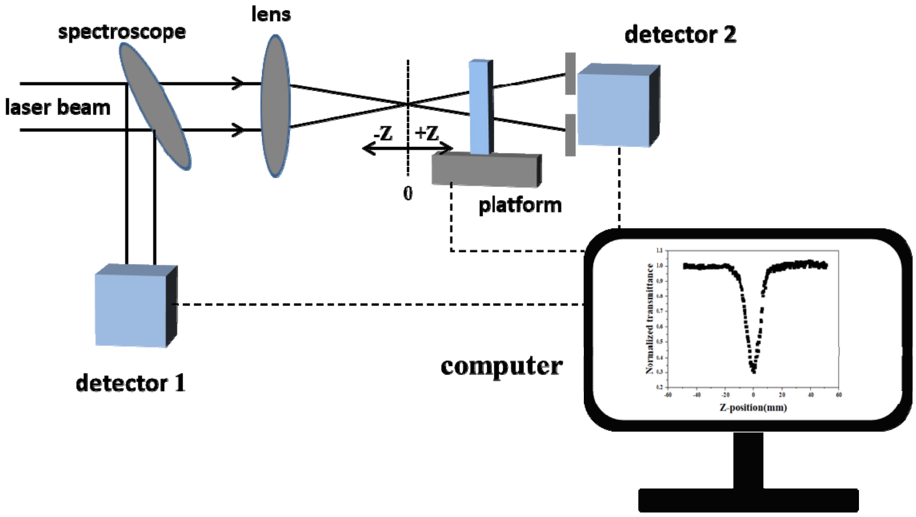

Figure S3 The schematic of Z-scan measurement.

Section S5 Z-scan test diagram of different concentration of Cu-bpy

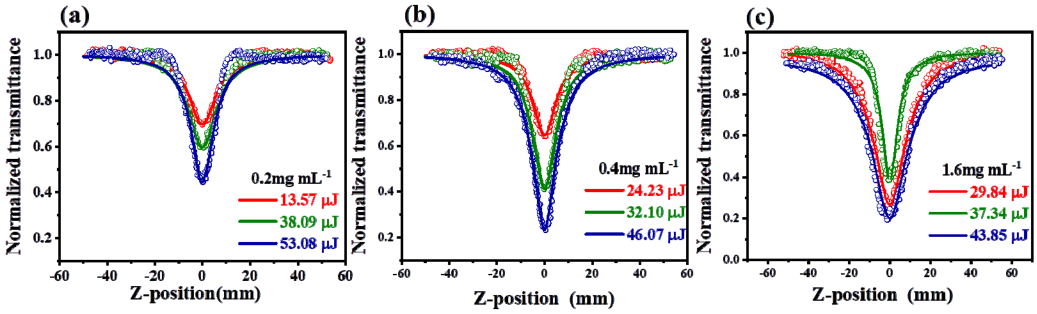

Figure S4. Z-scan curve of Cu-bpy with concentraion of 0.2 mg mL^{-1}, 0.4 mg mL^{-1} , 1.6 mg mL^{-1} in ethanol.

Section S6 DFT calculations for the electronic structures of Cu–BPY

The band gaps were calculated using the B3LYP functional, which includes a dispersion correction. Figure S5 shows the total and partial density of states of Cu-bpy.

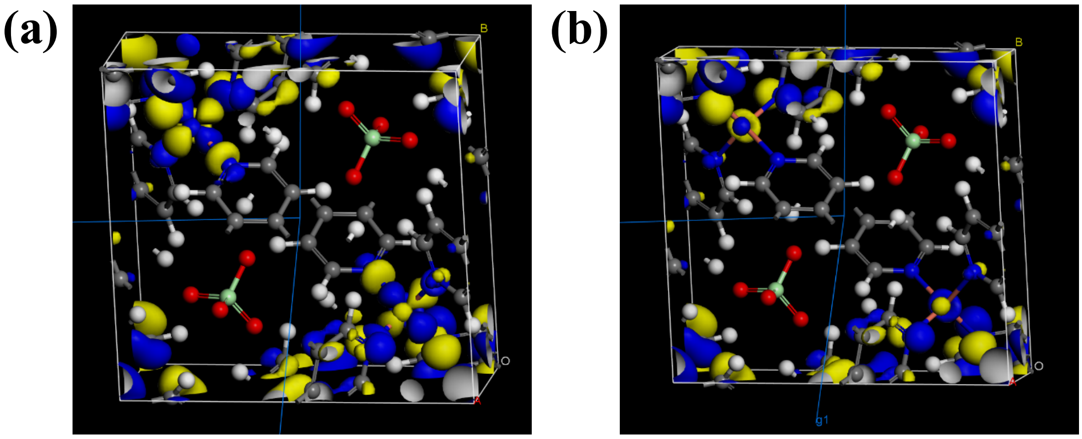

Figure S5. View of (a) HOMO and (b) LUMO for the profiles based on the DFT calculations of Cu–bpy.
